# What is the evidence that tau pathology spreads through prion-like propagation?

**DOI:** 10.1186/s40478-017-0488-7

**Published:** 2017-12-19

**Authors:** Amrit Mudher, Morvane Colin, Simon Dujardin, Miguel Medina, Ilse Dewachter, Seyedeh Maryam Alavi Naini, Eva-Maria Mandelkow, Eckhard Mandelkow, Luc Buée, Michel Goedert, Jean-Pierre Brion

**Affiliations:** 1 University of Southampton, Biological Sciences, Faculty of Natural and Environmental Sciences, SO17 1BJ Southampton, UK; 2 Univ. Lille, Inserm, CHU-Lille, UMR-S 1172, LabEx DISTALZ, 59000 Lille, France; 3 Department of Neurology, MassGeneral Institute for Neurodegenerative Disease, Massachusetts General Hospital, Harvard Medical School, Charlestown, Massachusetts USA; 4 Network Center for Biomedical Research in Neurodegenerative Diseases (CIBERNED), Madrid, Spain; CIEN Foundation, Queen Sofia Foundation Alzheimer Center, Madrid, Spain; 5 Dementia Research Group, BioMedical Research Institute, Hasselt University, 3500 Hasselt, Belgium; 6 Institut de Biologie Paris Seine-Laboratoire Neuroscience Paris Seine INSERM UMRS 1130, CNRS UMR 8246, UPMC UM 118 Université Pierre et Marie Curie, Paris, France; 7 DZNE (German Ctr. Neurodegen. Diseases), Bonn, Germany; 8 CAESAR Research Center, Bonn, Germany; 9 DESY, Hamburg, Germany; 10 MRC Laboratory of Molecular Biology, Francis Crick Avenue, CB2 0QH Cambridge, UK; 11 Laboratory of Histology, Neuroanatomy and Neuropathology Université Libre de Bruxelles, Faculty of Medicine, ULB Neuroscience Institute (UNI) 808, route de Lennik 1070, Brussels, Belgium

**Keywords:** Alzheimer's disease, tau, prion-like propagation, transmission, tauopathies, aggregation, seeding

## Abstract

Emerging experimental evidence suggests that the spread of tau pathology in the brain in Tauopathies reflects the propagation of abnormal tau species along neuroanatomically connected brain areas. This propagation could occur through a “prion-like” mechanism involving transfer of abnormal tau seeds from a “donor cell” to a “recipient cell” and recruitment of normal tau in the latter to generate new tau seeds. This review critically appraises the evidence that the spread of tau pathology occurs via such a “prion-like” mechanism and proposes a number of recommendations for directing future research. Recommendations for definitions of frequently used terms in the tau field are presented in an attempt to clarify and standardize interpretation of research findings. Molecular and cellular factors affecting tau aggregation are briefly reviewed, as are potential contributions of physiological and pathological post-translational modifications of tau. Additionally, the experimental evidence for tau seeding and “prion-like” propagation of tau aggregation that has emerged from cellular assays and in vivo models is discussed. Propagation of tau pathology using “prion-like” mechanisms is expected to incorporate several steps including cellular uptake, templated seeding, secretion and intercellular transfer through synaptic and non-synaptic pathways. The experimental findings supporting each of these steps are reviewed. The clinical validity of these experimental findings is then debated by considering the supportive or contradictory findings from patient samples. Further, the role of physiological tau release in this scenario is examined because emerging data shows that tau is secreted but the physiological function (if any) of this secretion in the context of propagation of pathological tau seeds is unclear. *Bona fide* prions exhibit specific properties, including transmission from cell to cell, tissue to tissue and organism to organism. The propagation of tau pathology has so far not been shown to exhibit all of these steps and how this influences the debate of whether or not abnormal tau species can propagate in a “prion-like” manner is discussed. The exact nature of tau seeds responsible for propagation of tau pathology in human tauopathies remains controversial; it might be tightly linked to the existence of tau strains stably propagating peculiar patterns of neuropathological lesions, corresponding to the different patterns seen in human tauopathies. That this is a property shared by all seed-competent tau conformers is not yet firmly established. Further investigation is also required to clarify the relationship between propagation of tau aggregates and tau-induced toxicity. Genetic variants identified as risks factors for tauopathies might play a role in propagation of tau pathology, but many more studies are needed to document this. The contribution of selective vulnerability of neuronal populations, as an alternative to prion-like mechanisms to explain spreading of tau pathology needs to be clarified. Learning from the prion field will be helpful to enhance our understanding of propagation of tau pathology. Finally, development of better models is expected to answer some of these key questions and allow for the testing of propagation-centred therapies.

## Introduction

The sequential appearance of tau pathology in the brains of Tauopathy patients has traditionally been considered to arise due to differential vulnerability of susceptible brain regions to disease processes, which is then reflected in the stereotypical progression of lesions throughout the brain. Recent evidence challenges this view and promotes the idea that tau pathology spreads through the brain using a prion-like mechanism. During the first EUROTAU meeting (Lille, France, April 2017 (http://lucbuee.fr/crbst_10.html), a round table discussion critically appraised this evidence and reflected on its clinical relevance. This review summarises that debate and makes recommendations that were suggested for clarification and identification of key-points for future studies. Additionally it was noted that various terms are used to describe tau pathology and that this can lead to confusion. Defining these terms would clarify their meaning and therefore standardise their use in future publications.

## Key definitions

There are numerous terms that are commonly used to describe aspects of tau pathology. These are listed and defined in Table 1 together with recommendations for consistent usage in future publications.

**Table 1:** Terminology for main tau assemblies and definition criteria

Name	Definition	Structural criteria	Molecular criteria
Tau pathology	Broad term designing abnormal molecular changes of normal tau as well as morphological changes.	Mislocalization of tau and/or pathological tau assembly in inclusion or aggregate.	Post-translational modifications of tau. Tau insolubility.
Tau inclusion	Morphologically distinct subcellular structure inside a cell.	Microscopically visible structure. Made of tau aggregates.	Properties of tau aggregates.
Tau aggregate	Assembly of tau into oligomers, fibrils, filaments, and NFT	Molecular tau assembly based on highly ordered ß-sheet structure.	Positive with ß-sheet (amyloid) sensitive dyes (Thioflavine T, Congo Red, LCOs). Tau hyperphosphorylation
Tau seed	A tau species inducing aggregation of tau	Molecular tau assemblies of various size providing a template	Positive with ß-sheet sensitive dyes
Liquid coacervates of tau	Membraneless organelles in a state of Liquid-liquid phase separation	Coacervation of tau into liquid droplets	Can acquire ß-sheet structure.
Tangles	Neuronal tau inclusions in somata	Composed of bundles of PHFs and SFs. Gallyas and Campbell-Switzer positive.	3R and 4R tau positive in AD
Neuropil threads	Tau inclusions in nerve cell dendrites	Composed of bundles of PHFs and SFs. Gallyas and Campbell-Switzer positive.	3R and 4R tau positive in AD
Dystrophic neurites	Axons forming the neuritic corona of plaques	Nerve cell processes in contact with Aß deposits. Some of them contain PHFs and SFs and are Gallyas and Campbell-Switzer positive.	3R and 4R tau positive in AD
Argyrophilic grains	Neuronal granular tau inclusions in dendrites	Tau filaments. Gallyas positive. Campbell-Switzer negative.	4R tau positive in AGD
Pick bodies	Spherical tau inclusions in nerve cell somata	Filamentous and vesicular material. Gallyas-negative. Campbell-Switzer positive.	3R tau positive in Pick disease.
Oligodendroglial coiled bodies	Tau inclusions in cell bodies of oligodendrocytes	PHF/SF like filaments. Gallyas positive. Campbell-Switzer negative.	4R tau positive in PSP and CBD
Globular oligodendroglial inclusions	Globular oligodendroglial tau inclusion	Gallyas positive.	Mainly 4R tau positive in GGTs
Tufted astrocytes	Astrocytes with thin and long radial processes containing tau inclusions	Tau filaments in cytoplasm and proximal portions of astrocytic processes. Gallyas positive. Campbell-Switzer negative.	4R tau positive in PSP
Astrocytic plaques	Astrocytes containing tau inclusions in a corona-like arrangment	Tau filaments in distal portions of astrocytic processes. Gallyas positive. Campbell-Switzer negative.	4R tau positive in CBD
Thorn-shaped astrocytes	Astrocytes with thorn-shaped processes containing tau inclusions	Spine-like perinuclear tau filaments. Gallyas positive.	4R tau positive in ARTAG

*AD* Alzheimer’s disease, *AGD* Argyrophilic grain disease, *ARTAG*, Ageing-related tau astrogliopathy, *CBD* Corticobasal degeneration, *GGTs* Globular glial tauopathies, *PSP* Progressive supranuclear palsy, *LCOs* Luminescent conjugated oligothiophenes

Each of the various tau inclusions is positive with some LCOs

A common feature of all tau assemblies is their immunoreactivity with tau antibodies, although peculiar tau epitopes can distinguish between them. For more details on specific tau inclusions, tauopathies, and silver staining properties see [9, 54, 86, 131]

## Round table discussion and questions for tau research

### Tau aggregation

#### Mechanisms of tau aggregation

Six tau isoforms are expressed in adult human brain, differing by the presence of 0, 1, or 2 amino-terminal inserts, and the inclusion or not of an amino acid repeat in the carboxy-terminal half [57]. Amino-terminal inserts are encoded by exons 2 and 3, with exon 3 never being expressed without exon 2, and the carboxy-terminal insert is encoded by exon 10. Assembled tau proteins are the molecular components of neurofibrillary tangles found in AD [18]. The assembly of monomeric tau into higher-order molecular species leads to the formation of tau filaments composing these neurofibrillary tangles. This ordered assembly must underlie tau seeding and recruitment of normal tau by pathological tau species to form aggregates made of filaments. Progressive formation of these filamentous tau aggregates is associated with insolubility (a biochemical definition) but insoluble tau may or may not be composed of filaments. However this information is not always provided in publications. These aggregates form different types of tau inclusions: e.g. neurofibrillary tangles and neuropil threads in AD and other tauopathies, argyrophilic grains in argyrophilic grain disease (AGD), oligodendroglial coiled bodies in tauopathies, astrocytic tufts in progressive supranuclear palsy (PSP), astrocytic plaques in corticobasal degeneration (CBD), Pick bodies in Pick disease, etc. (for review see [9, 85]).

**Fig. 1:**
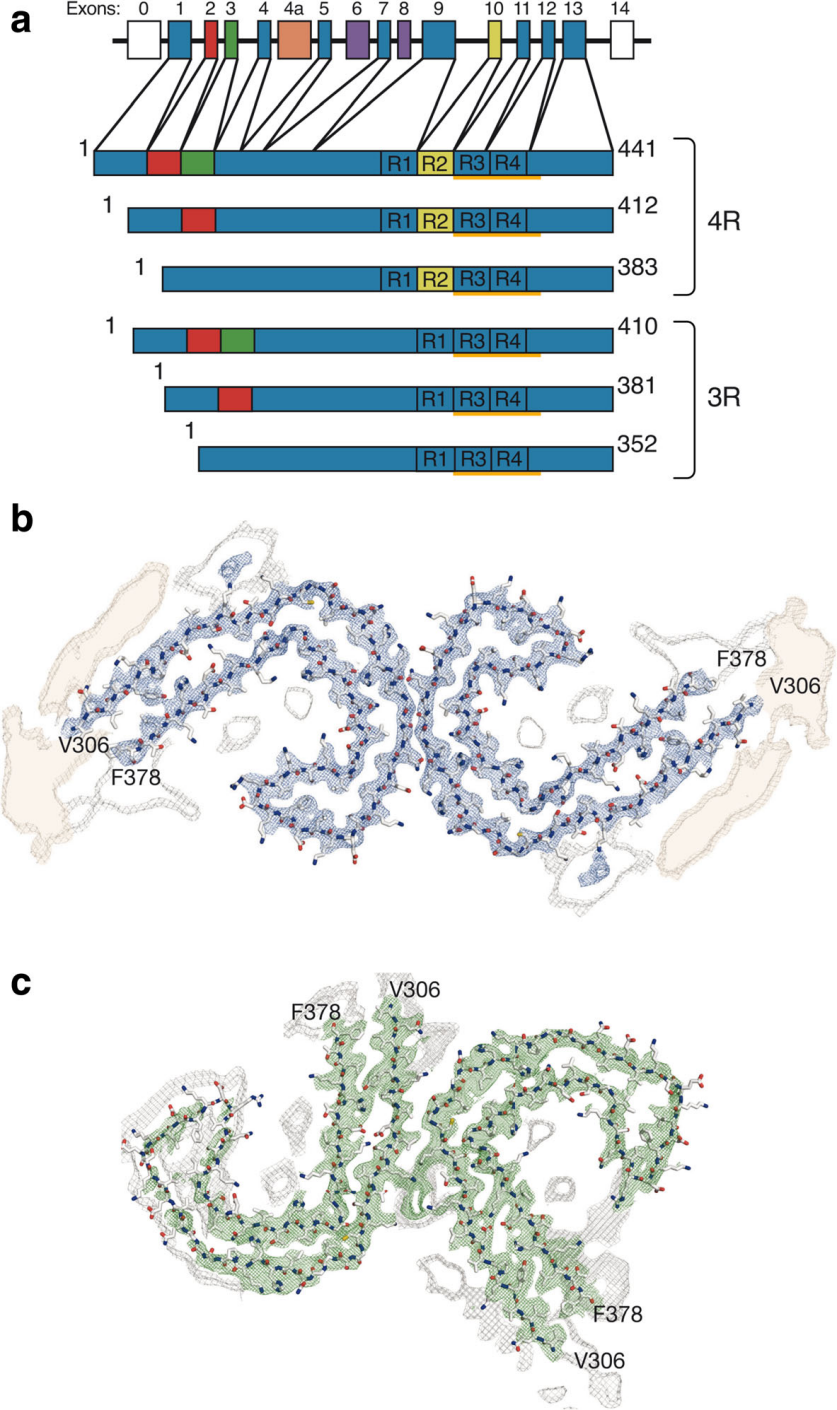
Human brain tau isoforms and the cores of tau filaments from Alzheimer’s disease. **a** MAPT and the six tau isoforms expressed in adult human brain. MAPT consists of 16 exons (E). Alternative mRNA splicing of E2 (red), E3 (green) and E10 (yellow) gives rise to the six tau isoforms (352–441 amino acids). The constitutively spliced exons (E1, E4, E5, E7, E9, E11, E12 and E13) are shown in blue. E0, which is part of the promoter, and E14 are noncoding (white). E6 and E8 (violet) are not transcribed in human brain. E4a (orange) is expressed only in the peripheral nervous system. The repeats (R1-R4) are shown, with three isoforms having four repeats each (4R) and three isoforms having three repeats each (3R). The core regions of the tau filaments from AD brain (V306-F378, using the numbering of the 441 amino acid tau isoform) are underlined. **b**, **c** Cross-sections of the cryogenic electron microscopy (cryo-EM) densities and atomic models of the cores of paired helical (**b**, in blue) and straight (**c**, in green) tau filaments. Each filament core consists of two identical protofilaments extending from V306-F378 of tau, which are arranged base-to-base (**b**) or back-to-base (**c**). The cryo-EM maps of the filament cores are at 3.4–3.5 Å resolution. Unsharpened, 4.5 Å low-pass filtered density is shown in grey. Density highlighted with an orange background is reminiscent of a less-ordered β-sheet and could accommodate an additional 16 amino acids, which would correspond to a mixture of residues 259–274 (R1) from 3R tau and residues 290–305 (R2) from 4R tau. Adapted from [46]

A lot of information is now available about which domains are needed for assembly of tau into filaments, and about the molecular arrangement of tau into paired helical (PHFs) and straight filaments (SFs). Assembly into filaments needs the tau repeats that form part of the core of, with the N-terminal half and the C-terminus extending outside to form the “fuzzy coat” [56, 139]. Hexapeptides in repeats 2 and 3 are needed for the induced aggregation of recombinant tau into filaments [134]. High-resolution structures of tau filaments from AD brain have been determined by cryogenic electron microscopy. The cores of PHFs and SFs from AD brain consist of two identical protofilaments extending from residues V306 to F378, which are arranged base-to-base and back-to-base, respectively (ultrastructural polymorphs) – illustrated in Fig. 1. The protofilaments probably define the seeds for tau aggregation. Each protofilament comprises eight beta-sheets, which adopt a combined cross-beta/beta-helix structure [46].

Oligomeric assembly of tau leads to the formation of soluble, non-filamentous species that are present in the brains of AD patients and some mouse models [88]. The accumulation of these oligomers might precede the formation of tau filaments, but their systematic contribution to the formation of filamentous insoluble aggregates in tauopathies needs further investigation.

The term “aggregation” is often meant to imply self-assembly of tau molecules into a filamentous form containing beta-pleated sheet structure similar to Alzheimer PHFs and SFs, but this is often not shown. Therefore the build-up of tau in the cell in various models of tauopathy should be described by a more neutral term, such as accumulation, if this information is not available.

What triggers tau seeding and aggregation in neuronal cells in vivo is still unclear. Tau inclusions in tauopathies have been observed to be associated with many other molecules that might play a role in promoting tau aggregation. Recombinant tau assembly can be triggered in vitro by heparin and other sulphated glycosaminoycans, RNA, as well as arachidonic acid [58, 68, 81, 138]. The strongly negative charges on each of these species (heparin, dextran sulphate, arachidonic acid and RNA) are likely to act as critical motifs for interaction with tau in vivo.

Tau may be involved in phase separation, with droplets forming through an interaction between the positively charged microtubule-binding domains and negatively charged molecules [5, 147]. Beta-sheet structures have been detected in these membraneless droplets. However, it remains to be seen what these in vitro findings mean for the aggregation of tau in vivo. The accumulation of non-filamentous tau should not be equated with tau aggregation. Local tau accumulations, not proven to be tau filaments, should not be confounded with tau aggregates. This is in agreement with observations indicating that cell stressors and signalling mechanisms can induce cellular accumulations of tau [61].

The initial transformation of normal monomeric tau into an abnormal tau seed is still a poorly understood event. A spontaneous, energetically favourable, acquired or inherited conformational change is a possibility. The growth of filaments by addition of tau species might rely on different mechanisms such as templated assembly or nucleated seeding. These are discussed in later sections of this review.

The molecular size of the tau assemblies that have the highest seeding efficiency when added to cultured cells, or injected in animal models, is still the subject of investigation. These studies are discussed in later sections of this review.

#### Assembly of different tau isoforms

Significant information is available on the relative incorporation of different tau isoforms in tau inclusions in different tauopathies. E.g. 3R and 4R tau isoforms accumulate in NFTs in AD, 4R tau accumulates in tau inclusions in PSP, CBD, AGD and 3R tau accumulates in tau inclusions in Pick disease. *MAPT* mutations give rise to tau inclusions made of either 3R + 4R tau (V337M, R406W), 3R tau (G272V, deltaK280) or 4R tau (P301L, P301S and all intronic mutations) [35]. Tau filaments have varying morphologies in these inclusions, reflecting (but not always) their tau isoforms composition [19, 55]. In vitro experiments indicate that 4R tau has a greater aggregation propensity than 3R tau [2], potentially underlying a mechanism by which pathological 4R tau species might assemble preferentially in vivo in 4R tau filaments. In vitro 4R tau can drive 3R tau aggregation and the presence of exons 2 and 10 promotes tau aggregation, whereas exon 3 depresses it [149]. 4R and 3R tau isoforms (with different N-terminal inserts) are however both expressed in human tauopathies and why 4R tau or 3R tau species start a selective assembly in vivo needs further studies.

In 4R tauopathies like PSP, AGD and CBD, 4R tau inclusions develop significantly in astrocytes (astrocytic tufts, astrocytic plaques) and in oligodendrocytes (coiled bodies) and the reason for the susceptibility of these glial cells (in addition to neurons) to develop tau aggregates in these tauopathies needs further research.

#### Role of tau phosphorylation and other posttranslational changes in aggregation

Tau is a phosphoprotein containing 85 potential serine, threonine and tyrosine phosphorylation sites and tau phosphorylation physiologically modulates microtubule assembly [9]. Soluble tau in biopsy-derived material purified from normal brain is phosphorylated but tau de-phosphorylation occurs rapidly post-mortem in autopsy-derived material from both human [92, 117] and rodent brains [98]. Tau phosphorylation is affected in several physiological conditions; e.g. foetal tau is more highly phosphorylated than adult tau and tau phosphorylation is higher during hibernation [10] and is increased during hypothermia [104]. In contrast, tau in aggregates in tauopathies is always hyperphosphorylated and this hyperphosphorylation is persistent even after long post-mortem delays.

Various groups have recently established lists of post translational modifications (PTMs) highlighted in different models (murine, human), including the Hanger group (https://docs.google.com/spreadsheets/d/1hGYs1ZcupmTnbB7n6qs1r_WVTXHt1O7NBLyKBN7EOUQ/edit#gid=0), the Mucke group [96] and the Kuret Group [47]. A study comparing wild-type mice and transgenic mice with abundant Abeta deposits in the absence of filamentous tau inclusions showed similar PTMs of tau, unlike what is seen for filamentous tau in AD [96]. Additional studies of animal models with filamentous tau inclusions would be informative. Meanwhile, it is possible to analyse the literature by focusing on the PTMs of tau in AD brains that are absent from control brains.

Phosphorylation is undoubtedly the most prominent PTM in AD brains and there are some phosphoepitopes present exclusively in AD brains and not found in normal healthy biopsy brain. For instance, phospho-Ser422 is found in different Tauopathies including AD but not in healthy biopsy-derived brain materials [20, 67]. Some antibodies also recognize conformational epitopes specific to tau assemblies. In vitro, phosphorylation is not a prerequisite for tau aggregation since tau assembly can be achieved with recombinant tau in the presence of different fibrillation inducers [24, 58, 103]. Nonetheless it can influence aggregation as was shown recently whereby prephosphorylated recombinant mutant tau proteins were used as an aggregation inducers and enabled the in vitro aggregation of recombinant mutant tau proteins [37]. Furthermore, equivalent tau seeding behaviour has been demonstrated for both non-phosphorylated and phosphorylated P301S recombinant tau proteins generated in vitro [45]. Intracerebral injection of preformed fibrils made of non-phosphorylated recombinant mutant tau enhances and accelerates propagation of tau pathology in mouse models expressing the same tau isoform [75, 102, 124]. Intriguingly though, endogenous tau seeded by these injections then propagates phosphotau epitopes. Hyperphosphorylation of tau might nevertheless play a role in modulating propagation of tau pathology since dephosphorylation of AD-derived hyperphosphorylated tau strongly reduced propagation after intracerebral injection in mice expressing wild-type tau [73].

Other PTMs include ubiquitination, acetylation, methylation, truncation, sumoylation, nitration, oxidation, glycation, glycosylation, etc. [66].

Altogether, in addition to phosphorylation, some PTMs on specific residues including methylation (Lys163, Lys174, Lys180 and Lys254), acetylation (Lys280) and nitration (Tyr29) might be linked to tau aggregates [47, 78, 109, 130]*.* Nevertheless, it should be noted that acetylated tau is associated with many tau inclusions in tauopathies [77] but acetylation of tau has been also reported to inhibit tau phosphorylation and its aggregation [30]. This is also true for phosphorylation, when for instance, KXGS sites are phosphorylated [115].

Accumulation of N- and C-terminally truncated forms of tau has been observed in several tauopathies [141, 150], and some of them can be generated by caspase [49] or calpain proteolysis. In vitro studies indicate that truncated tau might be more prone to aggregation than full-length tau. Native tau filaments extracted from AD brain (bearing all posttranslational modifications) efficiently induce endogenous tau seeding both in cultured cells [45], and after intracerebral injection in tau transgenic mice [79] and in wild-type mice [12]. Interestingly, such native filaments are more efficient at inducing seeding than preformed filaments (PFFs) [45, 65], although it is not clear which posttranslational or conformational changes confer this increased efficiency. This might be related to the reported increased resistance to proteinase K digestion and guanidine hydrochloride solubilisation of recombinant tau aggregates compared to tau aggregates from transgenic mouse brains [45], suggesting that more stable tau aggregates have lower seeding activity.

Altogether, these data indicate that (i) there is hyperphosphorylation in tauopathies and (ii) new phosphorylation sites and/or conformational epitopes, referred to as abnormal phosphorylation and/or conformation, are encountered in tau assemblies in tauopathies. (iii) Other PTMs are also present in these tau assemblies.

### Recommendations and cautionary notes:

1. Reports should be clearer when describing pathological tau in their models so that the detection of certain phosphotau epitopes is not confounded with hyperphosphorylation or aggregation. A more neutral term could be “abnormal phosphorylation” and the term “aggregation” in relationship with phosphorylation should only be used when genuine aggregates have been observed.
2. It is evident from the literature discussed above that interpretation of PTMs assigned to tau in both physiological and pathological conditions is tricky because such changes are crucially dependent on *post mortem* delay, a factor that is not always taken into consideration. This should be stated and accounted for in future reports. Ideally future studies investigating PTMs of tau would benefit from the inclusion of comparative elements in physiological situations in order to determine whether the modification described is a *de novo* pathological PTMs or the exacerbation of a physiological PTMs.
3. Analyses of PTMs of tau in murine models are not necessarily transposable to humans since the enzymatic content ensuring these PTMs is different.

#### Impact of tau mutations and tau levels on tau aggregation

*MAPT* mutations in familial tauopathies have a primary effect at the protein level or affect alternative splicing of tau pre-mRNA. These mutations have various functional effects: they can reduce in vitro the ability of tau to promote microtubule assembly or promote tau assembly into filaments and different types of tau inclusions are found in neurons and glial cells. Many frontotemporal dementia with parkinsonism-17 (FTDP-17) exonic mutations were shown to accelerate tau aggregation in vitro and their overexpression in transgenic animals is currently used to generate models that develop tau aggregates with aging. These models have proven to be very useful for analysis of cell-autonomous pathological cellular mechanisms associated with development of tau aggregates [39]. It seems more difficult to assess the respective roles of cell-autonomous and cell non-autonomous mechanisms in propagation of tau pathology in these mutant tau models compared to propagation in wild-type models [12, 40], since in the latter propagation is not dependent on the expression of an aggregation-prone mutant tau generating cell aggregates independently of seeding by propagation. Future studies should explore this to understand the differential contributions of cell–autonomous and non-autonomous mechanisms to spread of tau pathology. Some exonic or intronic tau mutations result in imbalance in 4R/3R tau ratios (with most often excess of 4R tau) [74, 120] without affecting total levels of tau. The in vivo mechanism by which such an imbalance favours tau aggregation is not clearly understood.

Microduplication of 17q21.31, which encompasses *MAPT*, has been reported to cause an AD-like phenotype with increased tau mRNA [89], but more studies are required to establish the pathogenicity of *MAPT* duplication. Overexpression of tau in FTDP-17 models is non-physiological and this should be carefully considered when analysing mechanisms of tau aggregation and modulation of tau aggregation. Interestingly, tau aggregation was not observed in a P301L tau knockin mouse expressing tau at physiological levels [51]. Transgenic animal models overexpressing full-length wild-type tau might develop a pathological phenotype but do not however systematically develop tau filaments [17, 62, 123]. Tangle formation from wild-type human tau has been reported in one rodent model in which all six isoforms of wild-type human tau were expressed on a null rodent tau background [8]. This implies that an interplay between the various tau isoforms plays a role in the mechanism by which they aggregate.

**Recommendation:** The respective roles of presence or absence of tau mutations, levels of expression of tau species, tau isoforms expressed in different animal models need to be carefully interpreted before considering tau aggregation mechanisms in these models validated for human diseases.

#### Role of other neurodegenerative-related proteins in tau aggregation

The simultaneous occurrence of several types of protein inclusions made of different aggregate-prone proteins characteristic of proteinopathies (alpha-synuclein (α-syn), TAR DNA binding protein-43 (TDP-43), Abeta peptide (Aß)) in tauopathies and other neurodegenerative diseases has been observed regularly [121], suggesting that they might mutually interact. E.g. there is experimental evidence that injection of aggregated Aß in mutant tau transgenic mice [15, 63] promotes tau pathology at the site of injection, but also in regions remote from the injection site, reminiscent of homotypic tau-seeded tau aggregation. Crossing between mutant amyloid precursor protein (APP) and tau mice [69, 90] enhances spreading of tau aggregation [108]. The mechanisms behind this enhancement might reflect cross-seeding phenomena. Cross-seeding of tau with Aß and increased tau spreading after injection of Aß-seeded tau have been observed [133]. Similarly cross-seeding between α-syn and tau has been demonstrated in cell free assays, and pre-aggregated α-syn PFFs induced increased tau aggregation in primary neurons and in mutant transgenic mice in vivo [64]. The relevance of these findings for the in vivo initiation of tau aggregation and enhancement of tau seeding in tauopathies is still poorly understood but might be a critical issue. E.g. in AD, the spatial and chronological progression of tau and amyloid pathologies differs initially [16, 129] but their co-existence and development in advanced cases (especially in cortical areas) might reflect a cross-talk.

### What is the evidence for prion-like propagation of tau aggregates?

A key, defining feature of prion-like behaviour is the stable propagation of distinct misfolded protein conformations. To demonstrate that tau aggregates engage in such prion-like behaviour, evidence of cellular uptake, templated seeding and subsequent intercellular transfer of the ensuing newly formed aggregates to induce similar aggregation in recipient cells is required. This section presents the evidence to support this notion from cell and animal models of tauopathy, and discusses whether such prion-like propagation underpins the spread of tau pathology in the brains of tauopathy patients.

#### Evidence for tau-induced seeding: cellular uptake and induction of aggregation

Seeding is the induction of aggregation of soluble tau by abnormal tau. The first step in this process is uptake of tau seeds by cells and subsequent templated aggregation and conversion of non-aggregated tau within those cells; i.e. the induced tau aggregates should physically resemble the parent seeds and also be able to induce aggregation of non-aggregated tau. There is evidence that tau seeds fulfil some, but not all of these criteria, as discussed below.

**Fig. 2:**
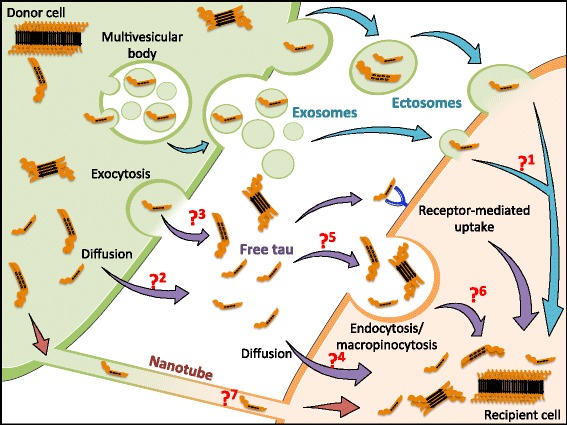
Transcellular transfer of tau: potential mechanisms underpinning this process. Tau proteins can be transferred from donor cells (green) to recipient cells (orange) using different routes. This figure highlights different pathways reported (blue or violet arrows) or hypothesized (red arrows) in the literature. Whether these pathways are used for physiological transfer of tau proteins to subserve as yet unknown functions of normal tau, or are pathological routes for transfer of tau seeds that can propagate transcellular transfer of tau aggregation, remains to be resolved. Pathway indicated by blue arrows - tau proteins are released in the medium by extracellular vesicles like exosomes and ectosomes. It is unclear how tau proteins carried inside vesicles reach the cytoplasm of recipient cells (Q1). Violet pathway- Around 90% of tau in the extracellular space is found as free protein. The mechanism(s) by which tau reaches the extracellular space in free form is unknown. Passive diffusion facilitated by a membraneous transporter/receptor (Q2) or active exocytosis (Q3) might contribute to this process. Uptake of free tau species by recipient cells, including HSPG or APP-mediated endocytosis/ macropinocytosis of tau accumulates have been reported. Whether free or aggregated tau is taken up by other mechanisms such as diffusion (Q4) or non-receptor mediated endocytosis/macropinocytosis (Q5) has not been resolved. Nor is it known how membrane-bound tau can escape from vesicles and enter the cytoplasm of recipient cells (Q6). Orange pathway- Tau was shown to be present inside nanotubes connecting cells in vitro and to allow its interneuronal transfer. This mechanism could potentially participate in prion-like propagation of tau pathology but whether it is a mode of transcellular transfer of seeding-competent tau species in vivo needs to be investigated

There is little doubt that aggregated tau can be taken up by cells through specific mechanisms (Fig. 2). Uptake of tau aggregates is by macropinocytosis ([45, 71] and requires heparan sulphate proteoglycans (HSPGs) [71]. Following uptake, tau seeds are in endosomes and must have access to the cytosol to induce aggregation of non-aggregated tau, and the latter mechanisms need to be explored.

Evidence for tau-induced seeding comes from numerous studies conducted in both cell and animal models (e.g. [25, 26, 45, 76, 83, 112, 142]. As outlined in Table 2, in these studies, tau seeds were derived from brain homogenates of tauopathy patients or symptomatic tau transgenic mice, cell and conditioned media from tau-aggregate bearing transfected cells, or generated from recombinant tau in vitro. Induction of aggregation was assessed using cell-based fluorescence assays [112, 142], biochemical insolubility assays [45] and immunohistochemical detection of disease-associated pathological tau inclusions [25, 26, 76]. In all instances tau aggregates were seed-competent, though to different degrees. This in itself may shed light on the mechanisms and factors promoting tau-induced aggregation in a pathological context. For example, native pathological tau aggregates derived from brain lysates exhibited 10 fold more seeding ability than equivalent aggregates generated in vitro [45].

**Table 2:** Different types of tau species that have shown seeding ability

Tau species	Derived from	seeding assay	Noteworthy features	Ref
P301S tau tg brain derived sarkosyl insoluble tau Recombinant P301S aggregates	P301S tau transgenic mouse brain (at symptomatic stages of disease) Heparin induced in vitro aggregation	Cell based P301S tau aggregation assay wherein insoluble tau inclusions formed within cells and were visualised by light microscopy and verified using biochemical insolubility assays. Sarkosyl insoluble P301S tau aggregates enriched in 30-50% sucrose fractions were verified by EM	1. Sarkosyl insoluble P301S tau from mouse brain has greater seeding capacity than total brain homogenate from P301S transgenic mice.	[45]
			2. Native (sarkosyl insoluble) P301S tau from mouse brain has greater seeding competence than recombinant P301S tau aggregates	[45]
			3. In vitro phosphorylation of recombinant P301S tau seeds does not increase their seeding competence.	[45]
			4. Seeding capacity of recombinant P301S tau becames equivalent to that of sarkosyl insoluble P301S tau from tg mouse brain when incubated with it in vitro.	[45]
			5. Sarkosyl insouble P301S tau from tg mouse brain separates into 30-50% sucrose fractions and comprises of AT8 and AT100 positive 6-10mer tau oligomers and short tau fibrils	[79]
AD brain derived sarkosyl soluble and insoluble tau	Fresh frozen AD brain homogenate (Braak stages 1-3)	FRET based cellular aggregation of CYP/RFP-tagged P301S-RD tau	1. In a significant number of cases, there was no biochemically evident insoluble tau (Braak stages 1-3) but the brain homogenates displayed strong seeding ability.	[48]
Recombinant P301S-RD tau oligomers and short fibrils	Heparin induced in vitro aggregation	Split luciferase based cellular aggregation of NLuc and Cluc-tagged P301S-RD tau	1. Tau trimers were smallest seed competent tau oligomers	[93]
AD brain derived tau oligomers	Fresh frozen AD brain	Split luciferase based cellular aggregation of NLuc and Cluc-tagged P301S-RD tau	1. Though tau oligomers extracted from AD brain ranging in size from *n* = 1 to *n* > 20, only oligomers equal to or greater than *n* = 3 exhibited seeding ability.	[93]
P301S tau tg brain derived undefined pathological tau species	P301S tau tg mouse brain homogenates (at pre-symptomatic and symptomatic stages)	FRET based cellular aggregation of CYP/RFP-tagged P301S-RD tau	1. Seeding activity detected as early as 1 month of age prior to emergence of misfolding (MC1 immunoreactivity which emerged at 3 m) or hyperphosphorylation (AT8 immunoreactivity which emerged at 6 m)	[71]
Recombinant RD-tau “strains” of distinct morphologies	Exposure of stably transfected cells expressing YFP-tagged P301L/V337M-RD tau to recombinant tau fibrils led to emergence of morphologically distinct tau inclusions; colonies of cells with the same inclusion were amplified and the relevant tau inclusion was stably propagated in a clonal fashion	Induction of morphologically distinct fluorescent accumulates of RD-tau evident by light microscopy following exposure to tau seed	1. Morphologically distinct tau strains were evident with different aggregation propensities and seeding abilities 2. Tau strains propagated morphologically distinct inclusions stably in cell culture and in vivo through successive generations	[112]
Recombinant 2N4R tau seeds Sarkosyl insoluble AD brain tau	Repetitive self seeded fibrilisation of recombinant 2N4R tau in vitro led to progressive increase in insoluble tau fibrils verified by EM AD-tau seed induced fibrillisation of recombinant 2N4R tau in vitro verified by EM	Induction of tau inclusions in rodent neurones NOT expressing exogenous human tau following exposure to tau seed Induction of tau inclusions in rodent neurones NOT expressing exogenous human tau following exposure to tau seed	1. Seeds derived from human AD brain (and therefore comprising of wild-type human tau) capable of inducing aggregation of wild-type rodent tau at physiological expression levels. 2. Induction of aggregation in cells verified by biochemical insolubility assays 3. Sarkosyl insoluble AD tau more seed competent than recombinant tau seeds generated in vitro but former able to confer its higher seeding competence to latter if seeded with it.	[65]

Tau seeds capable of inducing aggregation of soluble tau have been generated from recombinant tau in vitro, from cells stably transfected with tau fragments, from brains of transgenic mice, and from AD patients. This table lists some of these seeds from selected publications

Some studies also provide evidence that seeded tau aggregation is templated. Falcon et al. for example showed that native P301S tau seeds derived from transgenic mouse brains conferred their greater seeding competence to the less competent recombinant P301S tau seeds if co-incubated with them in vitro [45]. At the light microscopic level, tau aggregates induced in cells or in vivo have the same morphological appearance as the parent tau seed, and this also hints at a templated mechanism of conversion. This has been shown in many studies from the Diamond lab where morphologically distinct tau accumulates seed formation of accumulates that resemble the parent tau seed both in cell culture [112] and more recently in vivo [83]. That this may be relevant for tauopathies in humans has been demonstrated by the studies from the Goedert and Tolnay labs wherein injection of brain homogenates from different tauopathies into the brains of mice expressing non-aggregated human tau led to formation only of the corresponding tauopathy’s inclusions [26].

The evidence described above makes a strong case for tau aggregates engaging in prion-like seeding behaviour, but this is mostly derived from experimental models. To be certain that this is how aggregated tau behaves in human brain, one would need to demonstrate seeding activity in tau aggregates derived from tauopathy patients. Support for this has begun to emerge. Sarkosyl-insoluble PHFs extracted from AD brain tissues induce seeding in cultured cells and in wild-type mice [12, 65]. Moreover, tau seeds capable of inducing aggregation of non-aggregated tau in FRET based biosensor assays have been detected in brain homogenates and cerebrospinal fluid (CSF) of AD cases [48, 84] and in brain homogenates of Pick’s disease cases [111]. Thus there is circumstantial evidence that prion-like tau seeds are present in brain tissues in tauopathies and can promote tau aggregation.

#### Evidence for prion-like spread: Propagation of tau aggregates and their release

The studies discussed above make the case that tau seeds can induce further aggregation and thus exhibit some “prion-like” property. However, what is the evidence that such tau seeds engage in the other prion-like property, that of propagating their conformation? For this, one needs evidence of transfer of tau aggregates between cells, including secretion and uptake of seeds. Before discussing this evidence, one must explore the potential role, if any, of physiological tau release, since this too will require transcellular movement of tau.

#### Physiological tau secretion

There is much evidence that in physiological conditions, tau is secreted and found outside cells in the absence of cell death. This secretion appears to occur independently of tau pathology and may thus highlight a new function for tau. It is regulated by neuronal differentiation and activity [34, 41, 136, 144] and by the small GTPase Rab7A involved in the trafficking of endosomes, autophagosomes, and lysosomes [110]. In addition, *MAPT* mutations seem to impact tau release in vitro as well as in vivo [40, 82], strengthening the concept that wild-type tau associated with sporadic disease and mutant tau linked to FTDP-17 present different characteristics. Mechanisms underpinning tau secretion are still poorly understood but there are a number of cellular pathways implicated. At least 90% of tau is secreted in a free form [41, 136] but release is also mediated by vesicles such as exosomes [11, 119, 136] and ectosomes [41] (Fig. 2).

Tau is mostly secreted in a monomeric and/or truncated non-phosphorylated form [94, 105, 107] raising questions about the role of such secretion in the prion-like propagation of tau pathology. However, recent findings demonstrate that exosomes derived from N2A neuroblastoma cells overexpressing repeat domain delta-K280 fragments, contain tau aggregates and that these exosomes mediate tau aggregation in receiving cells [136]. In the same way, exosomes containing tau with seeding activity have been isolated from brains of tau transgenic mice, but not from biological fluids [106]. Tau in both free and vesicular forms has however been detected in CSF and interstitial spinal fluid (ISF) from both animal models [41, 143] and patients [48, 136] and in the latter this tau was seed competent.

These studies imply that tau in extracellular vesicles can act as a seed and therefore contribute to prion-like propagation of tau pathology but this has to be demonstrated in vivo. Additionally, whether secreted free tau can also act as a seed, has yet to be determined.

It is entirely conceivable that tau released in physiological conditions is not abnormally folded or otherwise pathological, and therefore cannot act as a seed for the propagation of tau pathology. Perhaps under pathological conditions, aggregated tau is released, which is capable of driving the transcellular propagation of pathology.

### Recommendation

Future studies should aim to decipher the mechanism(s) regulating tau secretion in physiological conditions, whether they are conventional or unconventional, and what role if any, they play in transcellular propagation of tau pathology in disease. In a pathological context, extracellular tau might also be related to clearance independently of any propagation/transfer to neighbouring/connecting cells. It is vital to take these parameters into account before designing therapeutic interventions targeting extracellular tau.

### Transcellular tau transfer

As discussed above, multiple studies in cell culture demonstrate that tau seeds can be released from donor cells and taken up by recipient cells where aggregation of non-aggregated tau is induced. Transcellular transfer of tau aggregates has been demonstrated between cells cultured serially in microfluidic chambers [142]. The spread of tau from neuron to neuron across trans-synaptic connections via exosomes, capable of seeding aggregation has been reported [106, 136]. Other mechanisms do not require secretion but a direct connection between cytoplasms. These structures, called nanotubes have been described in various cell types, including neuronal and immune cells. Recent papers showed that nanotubes support interneuronal transfer of tau fibrils in neurons [1, 128]. Whether such structures exist in the brain remains to be elucidated and whether the transfer of such seeds mediates aggregation in the recipient cells is still not demonstrated. These studies illustrate that there are many ways for tau to be secreted but it is not clear how efficient each of these is in terms of transfer of tau seeds. For instance though 90% of tau is secreted in a free form with only a minority being secreted from ectosomes, the latter are more efficient for transcellular tau transfer. This highlights the importance of understanding which mechanism(s) of trans-cellular tau transfer plays a role in physiology and which may become more prominent in pathology.

Only a few papers address the actual mechanism(s) regulating cellular tau uptake. Free tau has been shown to bind receptors on receiving cells, including HSPGs [71] and APP [125], which are proposed to enable cellular entry via receptor-mediated endocytosis. To date, nobody has investigated the mechanism(s) regulating vesicular tau uptake. Nevertheless, tau seems to be able to activate signal transduction pathways in receiving cells leading to Ca^2+^ release and neuronal damage [59, 60]. The latter studies highlight pathogenic effects of extracellular tau other than those required for prion-like behavior. Whether or not such effects of extracellular tau are involved in spread of tau pathology remains to be determined.

### Neuroanatomical spreading of tau aggregates

The cellular studies described above make a case for transcellular transfer of tau pathology. Supporting these findings, trans-synaptic propagation of tau pathology has been demonstrated using a variety of different approaches in transgenic mice. Pioneering research in this field, Clavaguera et al. showed not only the induction of tau aggregation in rodent brain following intracerebral injection of brain homogenates containing tau seeds, but also the time-dependent appearance of tau pathology in anatomically connected brain regions [25]. Others have also reported the appearance of tau pathology in areas connected to the sites injected with tau seeds or viral vectors expressing tau [3, 40, 76, 102]. De Calignon et al. [36] and Liu et al. [91] used a model in which expression of human tau was restricted to the entorhinal cortex only and yet tau pathology was evident in anatomically linked regions not expressing the human tau trangene. This could only have happened if tau seeds generated in the entorhinal cortex had propagated to induce aggregation in brain regions connected to the entorhinal cortex. Studies in tau transgenic mice indicate that tau seeds predict spread of disease by appearing in brain regions *prior* to appearance of any other pathological change [72]. Moreover, another study in a rodent model showed that if one of the hypothesised modes of transsynaptic transfer of seeds, exosome synthesis is inhibited, propagation of tau pathology is significantly suppressed [11]. Propagation of abnormal tau in a P301L model without expression of endogenous tau has been observed, suggesting that spreading of tau does not always require templated misfolding of endogenous tau [137].

Thus there is persuasive evidence to support the concept that seed competent tau aggregates can be taken up by cells, induce aggregation of non-aggregated tau and then get released by cells. This process might be repeated across synaptically linked networks and in this way tau pathology spreads through neuroanatomically linked brain regions. However, for this to be relevant to the mechanism by which tau pathology spreads in humans, one would need evidence that this occurs in the brains of patients. The fact that tau pathology spreads in a defined neuroanatomically connected pattern in AD, as described by the Braak staging is tentative evidence for trans-synaptic spread of pathology. However, the sequential appearance of tau pathology in anatomically connected regions may simply reflect a spatio-temporal vulnerability of different anatomical regions to tau aggregation, unrelated to propagation. A recent study reporting seeding behaviour in brains of AD patients argues against this [48]. In this report, seeding ability in different brain regions correlated positively with Braak stage, negatively with MMSE scores, and preceded overt tau pathology. This strongly implies that spread of tau pathology occurs by a prion-like trans-synaptic mechanism. It also explains the histopathological observation made by Duyckaerts et al. 20 years ago when the concept of prion-like propagation of non-prion protein aggregates was in infancy [42]. They reported the conspicuous absence of tau pathology in a frontal cortical region that had been anatomically disconnected from the limbic region as a result of neurosurgery decades before the patient developed AD. This was despite extensive pathology in immediately adjacent brain regions as well as in the limbic and isocorttical areas. Together these two studies in AD patients provide the most compelling evidence that prion-like propagation of tau pathology occurs in human brain and underpins the characteristic progression of pathology in tauopathies.

Thus collectively, there is ample, albeit circumstantial evidence to support the case that prion-like propagation contributes to the spread of tau pathology in tauopathies.

### Cautionary notes and recommendations

1. It must be noted here that prion-like behaviour should not be extended to the infectivity behaviour of prions. Prions induce templated misfolding of a normal prion protein, the propagation of this misfolding in the brain, across tissues (e.g. from periphery to brain) and between organisms. Prion-like behaviour of tau for now is mainly documented by templated seeding and propagation of aggregation across brain areas. Some animal experiments suggest however that an intracerebral tauopathy can develop after peripheral administration of tau aggregates [27]. Careful analysis of the different steps of these complex processes is needed for all molecules exhibiting prion-like behaviour and for differentiating them form infectious entities [43].
2. Some of the animal models that have thus far been used to demonstrate transcellular spread of tau seeds have limitations, which may confound the evidence that they provide and this should be considered. For example:

- Many models use intracereberal injections of tau seeds in the form of different brain fractions or recombinant tau. This material will diffuse some distance away from the injection site. Although this might result in uptake of tau seeds and intracellular seeding, it does not necessarily imply transcellular spread and propagation through neuroanatomically connected areas of aggregated tau. This should be taken into consideration when interpreting their data.
- The animal models that utilise tissue-specific transgene expression use drivers that have been reported to be leaky [146]. Even if this occurs to a very small extent, it gives rise to the possibility that some of the induction of aggregation evident in distal regions arose due to small amounts of transgene expression there rather than seed propagation from the tissue of greatest transgene expression.

#### Evidence for trans-synaptic and non-synaptic transmission of tau pathology

The studies described above make a strong case for the propagation of tau pathology to be mediated by synaptic mechanisms. However even in the prion field, where this concept emerged, there are indications that trans-synaptic propagation may not be the only means of spread of prions [116]. In the tau field too, it may be imperative to keep an open mind in this regard and entertain the idea that the spread of tau pathology through the brains of tauopathy patients may occur by a number of means including trans-synaptic spread, interstitial diffusion and even microglial intervention. To this end, one study has shown that microglial depletion or suppression of production by microglial cells of exosomes significantly reduces the propagation of AT8-positive oligomers in a rodent model [11]. There is precedence for glial cells playing a role in transcellular transmission of Huntingtin aggregates in a *Drosophila* model [101]. The demonstration of a similar contribution of glia in propagation of pathological tau suggests that glial cells may play a significant role in transcellular transfer of pathological aggregates in many proteinopathies.

Propagation through both anterograde and retrograde pathways between connected cells might occur, potentially through different mechanisms, and this needs further investigation.

#### The nature of the tau species that spread/ propagate

Tau seeds derived from multiple sources have been used and in all cases shown to be competent at inducing tau aggregation and transcellular propagation of aggregation. This includes seeds generated by incubating recombinant tau protein with fibrillization inducers in vitro, or from transfected neuronal and non-neuronal cell lysates and conditioned media, or obtained from brain homogenates of transgenic mice and human tauopathy brains. Tau seeds have also been generated in vivo following the injection of viral vectors expressing tau into rodent brain. Though the nature of the tau species may differ between these studies, and though there may be multiple tau species with seeding ability in human brain, one can infer the properties all seed-competent tau species have in common.

Various studies have shown, perhaps unsurprisingly, that monomeric soluble tau or mutant tau incapable of aggregation has no seeding ability [45, 93]. There are however conflicting reports as to the minimal number of tau units required to confer efficient seeding ability. Recent findings showed that monomeric tau (exhibiting unique structure with accessible VQIINK and VQIVYK motifs) derived by sonication from fibrillized tau, seeded aggregation of tau in cells (http://www.alzforum.org/news/conference-coverage/monomeric-seeds-and-oligomeric-clouds-proteopathy-news-aaic). One study reported that a minimum of 3 tau molecules is required [93], whilst another report only showed seeding when 6–10 tau molecules were aggregated [79]. Nonetheless both studies showed that larger oligomers comprising between 10 and 100 tau units display greater seeding competence [93]. In line with this study, others too have shown that tau oligomers can induce seeding, both in cell culture [136] and in vivo [22].

As well as for oligomers, seeding capability of larger insoluble tau aggregates has also been characterised. Jackson et al. [79] characterized the seed-competent native tau species from the brains of P301S tau mice, and showed that a range of short tau fibrils, including short filaments and ring-like structures, phosphorylated at the AT8 and AT100 sites, were endowed with the greatest seeding capacity [79].

The studies above have extensively characterised tau seeds generated often in experimental models. However, for this to be relevant to humans, future studies would need to compare and contrast these experimental tau seeds with those found in the brains of patients with different tauopathies. This has already begun, and early indications are that tau seeds found in AD brain can be isolated from both soluble and insoluble brain fractions [48]. This implies that there are multiple tau seeds in existence in diseased human brains that comprise both soluble and insoluble tau aggregates.

Summarising these studies, one can therefore conclude that monomeric/oligomeric/ multimeric tau species with a unique conformation might be the only requirement for initial tau seeding behaviour. Beyond this, other characteristics of the tau seed, such as how large it is, PTMs and how it is generated i.e. whether it is natively formed or generated in vitro, will determine seeding competence. Ascertaining which tau species are the key-“building blocks” of filaments is an issue. The nature of the conformation will also play a part with the general assumption that beta–pleated sheet content is a determinant for seeding capability. It will be important to identify and characterise the various tau seeds found in tauopathies and the factors that regulate seeding behaviour, if this is to be an avenue for preventing propagation of tau pathology in tauopathies.

### What is the evidence for “tau strains or conformers”?

Prion strains with distinct biological properties can explain the variations in clinical presentations and in neuropathological lesions in prion diseases [29]. Similarly, the notion that tau “strains” exist might explain peculiar patterns of neuropathological lesions in different tauopathies, and the intensity and the kinetics of disease progression [28]. A given strain may determine where the disease process starts, followed by prion-like spreading based on the connectivity of the primarly affected regions. Experimental injections of various types of brain extracts from patients with different tauopathies in animal models induce different patterns of tau pathology, cellular and neuropathological lesions [26, 83, 84]. The treatment of cultured cells with similar extracts induces the formation of different types of inclusions [112] and cell based assays to measure tau seeding activity will be helpful to characterize tau strains [84].

These observations are compatible with the existence of different tau strains. As for prions, different structural and biochemical features might define these strains. Distinct conformations evidenced by a relative resistance to proteolytic enzymes are such features. The definition of a molecular conformer might fundamentally be structural and future cryo-EM studies may well provide this information. There are likely to be different molecular conformers made of 3R + 4R, 3R and 4R tau isoforms. Protein sequence and mass spectrometric analyses have revealed that the protease-resistant core units of tau aggregates differ between tauopathies [127]. It also remains to be seen if different strains of aggregated tau exist within these categories, for instance between AD and chronic traumatic encephalopathy, or between PSP and CBD.

Many of the present studies were done at the light microscopy level and more work is needed to document the structural characteristics of tau strains in different tauopathies. Tau assemblies with distinct conformations that do not stably propagate their properties in vivo are probably not *bona fide* strains. Like for prions, it is expected that a defined tau strain will recapitulate similar patterns of neuropathological lesions and keep its biological properties after serial passage in animal models. Whilst this has been shown for some tau accumulates [83, 84, 112], it is not yet clear that it is a property shared by all seed-competent tau conformers.

### What is the evidence that propagation of tau aggregates is toxic?

#### Dissociation between aggregation, propagation and toxicity

The general assumption is that propagation of tau aggregates is synonymous with the propagation of toxicity. This is because tau aggregates are believed to be toxic, so one would assume that their induction and propagation would cause dysfunction and degeneration of the neuronal networks through which they spread. Supporting this, some studies have shown that seeding ability of different recombinant tau seeds correlates with their toxicity in vitro [112] and to some extent in vivo [83]. Another study has shown both electrophysiological deficits and resultant behavioural dysfunction following induction of templated tau seeding [124]. However, this has not been assessed in the majority of studies showing prion-like propagation of tau pathology in vivo*.* Degeneration of tangle-bearing neurons has been described when the tau seeds induced tangle-like pathology in neurons in the locus coeruleus after injection in this area [76]. Others clearly state that there was no degeneration despite clear propagation of tau aggregates [3, 137]. This is an area that requires further investigation because the relationship between propagation of tau aggregates and tau-induced degeneration is not clear. In the prion field too, the distribution of misfolded prion protein PrP^SC^ alone does not predict neurodegeneration [4].

Toxcity of tau independent of its aggregation is another caveat that should be considered. Tau interactions with other cell components can be toxic to some cellular process, and lead to the spreading of toxicity through signalling mechanisms.

#### Different pathological tau species employ different mechanisms of toxicity

Perhaps a lack of clarity arises because different pathological tau species might use different mechanisms of toxicity (Table 3). Soluble hyperphosphorylated tau species (which may be monomeric or small oligomeric aggregates) cause neuronal dysfunction characterised by breakdown of cytoskeletal integrity, disrupted axonal transport [100] and synaptic dysfunction in *Drosophila* [32, 97]. This is perhaps more accurately described as “phospho-tau mediated dysfunction” rather than overt toxicity. Tau oligomers and small tau fibrils, which are most likely to engage in transcellular propagation, are associated with toxicity arising from gain of toxic function mechanisms [53, 87]. Whether this is due to the transcellular propagation of aggregation is not proven and further work is required to understand the connection between these two phenomena. In contrast, the toxicity of larger insoluble oligomers and tangle-like structures is debatable with some studies stating that they are toxic whilst others implying that they are protective [14, 31, 33, 52, 114, 122, 140]. Clearly different pathological tau species are differentially toxic and which species forms at which time point in the neuronal circuit through which the tau pathology is spreading will determine tau toxicity. Moreover, within these neurons, different forms of tau could be responsible for templated propagation of pathology and tau-induced neuronal dysfunction.

**Table 3:** Potential modes of tau toxicity

Pathological change and Tau species implicated	Potential modes of tau toxicity	Selected References
Hyperphosphorylation (e.g. soluble monomer/dimer)	Loss Of microtubule-binding (and other) Function(s) (LOF) leading to axonal transport and synaptic defects reflected in mitochondrial clumping, Golgi disruptions and mis-sorting of synaptic proteins. Mis-localisation may also be evident causing Gain Of toxic Function (GOF). Collectively these may be responsible for neuronal dysfunction at early stages of disease. It is possible that a partial LOF is required for, and leads to an eventual GOF	[31, 32, 52, 97, 100]
Misfolding/aberrant folding and aggregation into small aggregates (e.g. sarkosyl soluble oligomers)	Neuronal dysfunction and neurodegeneration evident in some models in the absence of larger aggregates implying that smaller soluble oligomeric species responsable for these phenotypes	([53, 87])
Aggregation (into large insoluble oligomers such as granular tau oligomers and filaments including tangles)	Space-occupying lesions resulting in GOF. Toxicity debated because in some models rescue of neuronal dysfunction and degeneration evident despite persistence of larger aggregates.	([14, 31, 33, 52, 114, 122, 140])

The various pathologial changes in tau may be responsible for causing loss of normal function (LOF) or gain of toxic function (GOF). In the face of emerging novel functions of tau, there may be numerous modes of toxicity via a number of LOF mechanisms. Toxicity resulting from GOF mechanisms are more difficult to dissect but based on reports of neuronal dysfunction or neurodegeneration in the absence of large insoluble tau filaments, the tau species responsible (or not as the case may be) are begining to be understood. Recommendation: Future studies should seek to clarify terminology and consistency in ascribing modes of toxicity to tau species

### Recommendation

Terminology used to describe tau toxicity should be clarified and consistency sought. This will enable a fuller picture to be built to enable understanding of which pathological tau species (hyperphosphorylated monomer, oligomer, fibril) is responsible for which effect (dysfunction, degeneration, propagation).

### What is the role of genetic factors in the propagation of tau pathology?

Genetic and genome-wide association (GWAS) studies have identified risk factors for AD [132] and other tauopathies [70]. These genes are implicated in many different cellular processes but how they affect these processes is not known. Genes products involved in exocytosis and endocytosis, the function of synaptic vesicles, protein clearance, intracellular transport, etc. might be directly linked to the propagation of tau pathology [13]. Much experimental work is still needed to link the effects of these gene variants to increased or decreased propagation of tau pathology.

Some GWAS have identified direct (or indirect) binding partners of tau (APOE, BIN1, PICALM, CLU). Inheritance of the *APOEe4* is a well-established risk factor for AD and tau-mediated neurodegeneration is aggravated by APOE4 [118]. A reduction in insulin signaling in the brain is a feature of AD and APOE4 impairs neuronal insulin signaling [148]. BIN1 is a negative regulator of clathrin-mediated endocytosis and modulates tau toxicity [23]. The level of neuronal-specific BIN1 isoform is reduced in AD brain and lower levels of BIN1 promote propagation of tau pathology in cultured neurons [21]. PICALM, which is also involved in clathrin-mediated endocytosis, modulates autophagy and alters the clearance of tau [95]. PICALM levels are decreased and PICALM co-localizes with tau inclusions in AD [6] and in other tauopathies [7]. PTK2B, involved in a cell adhesion pathway, was also recently identified as a modulator of tau pathology [38].

*MAPT* mutations in familial tauopathies favour tau aggregation and determine which type of tau isoforms are included in tau aggregates (see also above paragraph on tau aggregation) [50]. Although this might facilitates propagation of tau pathology, distinguishing the role of cell autonomous mechanisms (e.g. “spontaneous” formation of tau aggregates in different cells independently of their connections) from cell non-autonomous mechanisms (e.g. dependent of transfer of pathological tau between connected cells) in tau spreading might be difficult.

## Future directions

### How can future studies enhance our understanding of propagation of tau pathology?

#### Need to better understand the role of prion-like propagation of tau pathology in human tauopathies

A fundamental question is whether prion-like mechanisms play a role in sporadic human tauopathies and other neurodegenerative diseases. Although prion-like mechanisms may also operate in familial forms of these diseases, they may not be required there, because all cells express the mutations. Further studies are needed to clarify if some cases of sporadic tauopathies can be acquired though it would require unusual conditions. A difference between sporadic tauopathies and prion diseases is the lack of evidence for acquired cases in the former. However, recent findings may begin to provide this evidence. Recent work, in the UK, on people who had received cadaver-derived human growth hormone as children, which was contaminated with prions (and possibly AD Aß seeds), developed a prion disease and showed some Aβ deposits at autopsy. However, they had no tau inclusions, nor did they suffer from AD [80]. In another series of French patients who died from iatrogenic Creutzfeldt-Jakob disease after the injection of cadaver-derived human growth hormone, some cases showed tau inclusions and tau and Aß contaminants were detected in batches of growth hormone (Duyckaerts et al., in press Acta Neuropathologica 2017).

The spreading of tau pathology in the brain during progression of tauopathies is also compatible with selective vulnerability of neuronal populations to pathological processes [135]. This could be true even in the framework of a prion-like mechanism. Connected cells might be differentially sensitive to prion-like mechanisms of release and uptake of aggregates, and to seeded aggregation. Independently or not from prion-like mechanisms, some cells could successfully degrade aggregates for a longer time before becoming affected. This may explain the frequently observed age dependence of these human diseases, reflecting the ability of cells to degrade aggregates and prevent spread. These proteins may have an intrinsic tendency to aggregate (although tau is very soluble) and only when the balance between degradation and aggregation favours the latter, will the ordered assemblies of tau be able to spread.

Thus, the respective roles of prion-like mechanisms and selective vulnerability in the development of tau pathology remain to be clarified.

#### Learn from the prion field

In the prion field different strains of PrP^SC^ are associated with different prion diseases. The “prion-like” behaviour of tau seeds has led to the concept that distinct tau strains underpin different tauopathies. The popularity of this idea is growing fast, and there is a large body of evidence to support many, though not all, of its aspects [135]. However, unlike the prion field where distinct strains of PrP^SC^ are evident in different prion diseases, and where degeneration is believed to require spread of PrP^SC^, this has to be more clearly demonstrated in tauopathies. Future studies need to identify distinct tau strains in different tauopathies and demonstrate that the spread of tau seeds is required for the propagation of pathology. Added to this, future studies need to focus on understanding the impact of seeding and propagating tau aggregation within affected neural networks to appreciate if this has any clinical significance. A large body of research in the prion field now suggests that the PrP^SC^ species responsible for transcellular propagation and neurotoxicity are partially distinct. Since multiple pathological tau species have been described, it is possible that the same is true for tauopathies. This needs further investigation.

Perhaps learning from the prion field will facilitate the studies proposed above. Even though tau seeds may be better described as “propagons” rather than *bona-fide* “prions”, the methods used to characterise and study PrP^SC^ will be invaluable for deciphering the “prion-like” properties of tau seeds. Such studies should enable better understanding of the templated seeding behaviour of tau strains, the mechanisms underpinning their internalisation, secretion and eventual transcellular spread, their mechanisms of toxicity and how tightly this is linked to their propagation, and the role of glial cells in their spread. It would also be important to determine the level of amplification of tau seeds during spread, which to date has not been addressed, and which can be assessed using experimental approaches routinely used in the prion field.

#### Understand physiological role of tau secretion and its relevance to pathological tau spread

As discussed here, tau can be found extracellularly but it is not clear if this is pathological or indicates an as yet unknown function of tau. Since the two key tenets of the prion-like spread of tau pathology are secretion and uptake of tau species, it becomes imperative to investigate how secretion and uptake of pathological tau seeds differ from secretion and uptake of normal tau. It can be hypothesized that if the tau species released are seed-competent, then this process could contribute to transcellular propagation of tau pathology. However future studies need to demonstrate this.

#### Developing better models to study tau aggregation and propagation

Models expressing human wild-type and FTDP-17 tau at normal physiological levels, tau knock-in models [52], models expressing the whole set of human tau isoforms [8] and models reproducing the exact alternative splicing of the human tau gene are promising approaches for the future (http://www.alzforum.org/news/conference-coverage/next-generation-mouse-models-tau-knock-ins-and-human-chimeras). Grafting human neurons derived from stem cells within animal models, simulating exposure to a complex 3D in vivo environment is another approach [44]. These second-generation models are expected to provide further insights into the physiopathology of tau aggregation and tau spreading.

### Propagation-centred therapies

The fact that AD brains and brain homogenates from tauopathy models contain tau seeds capable of inducing tau aggregation in vitro and this correlates with progression of disease state [72, 126], implies that anti-seeding approaches may have therapeutic value. Blocking of tau aggregation and inter-neuronal tau propagation in vitro by anti-tau antibodies has been reported [99]. Anti-tau antibodies capable of suppressing tau seeding activity in vitro effectively reduced tau aggregation in a transgenic model of tauopathy; however whether this was through reduction of propagation needs to be confirmed [145]. Spreading of tau pathology after injection of PFFs in mutant tau mice was significantly reduced by passive immunisation with phosphotau antibodies [113]. The most convincing evidence for a causal role of prion-like spread of tau aggregation in pathogenesis of disease will come if interference with propagation of tau pathology were to suppress neurodegeneration and lead to clinical benefits. Promising results are emerging from mouse models and they may soon translate to patients.

## Concluding remarks

The concept that brain spreading of tau pathology in tauopathy occurs by a “prion-like” mechanism is fast gaining popularity. The key tenets of this hypothesis are that diverse conformational tau strains exist in different tauopathies, and that they drive templated aggregation followed by the intercellular propagation of tau pathology. While the evidence in favour of this hypothesis is growing daily, its clinical relevance is still debated [135]. Furthermore, it is not clear how spread of tau pathology through this “prion-like propagation” relates to spread of pathology arising from differential spatio-temporal vulnerability of connected neuronal populations. Future studies which take into account some of the points raised here are needed to document the respective roles of these pathological mechanisms in tauopathies. Nonetheless, since clinical symptoms are likely to manifest only after extensive spread of pathology, understanding all potential modes employable by pathological tau to traverse neural circuits opens novel therapeutic avenues to arrest the spread of tau aggregates at a preclinical stage.
